# How do university students perceive the educational transition in the COVID‐19 era: A cross‐sectional study from 15 Arab countries

**DOI:** 10.1002/hsr2.1254

**Published:** 2023-05-09

**Authors:** Nael Kamel Eltewacy, Rana K. Abu Farha, Sajeda G. Matar, Afnan W. M. Jobran, Nesma Gebril Nagi, Amira Yasmine Benmelouka, Hagar Ismail Rohim, Enas M. Yasseen, Omnia Ayman, Aya N. El‐Din Sayed, Reem Ahmed M. Abdallah, Rana Ahmed M. Abdallah, Alaa M. Yousef, Ahmad B. Yahia, Muhamad Zakaria Brimo Alsaman, Mahmoud A. Ebada, EARG Collaborators

**Affiliations:** ^1^ Eltewacy Arab Research Group; ^2^ Faculty of Pharmacy Beni‐Suef University Beni‐Suef Egypt; ^3^ Faculty of Pharmacy Applied Science Private University Amman Jordan; ^4^ Faculty of Pharmacy University of Jordan Amman Jordan; ^5^ Faculty of Medicine Alquds University Jerusalem Palestine; ^6^ Department of Biostatistics, High Institute of Public Health Alexandria University Alexandria Egypt; ^7^ Faculty of Medicine University of Algiers Algiers Algeria; ^8^ EHS de Psychiatrie Cheraga Algiers Algeria; ^9^ Faculty of Medicine Beni‐Suef University Beni‐Suef Egypt; ^10^ Faculty of Pharmacy Nahda University Beni‐Suef Egypt; ^11^ Pharmaceutics department, Faculty of pharmacy Nahda university Benisuef Egypt; ^12^ Faculty of Medicine Al Balqa' Applied University Salt Jordan; ^13^ Faculty of Medicine Hashemite University Zarqa Jordan; ^14^ Department of Vascular Surgery Al‐Razi Hospital Aleppo Syria; ^15^ Faculty of Medicine Zagazig University Zagazig Egypt; ^16^ Resident Physician, Egyptian Fellowship of Neurology Nasr City Hospital for Health Insurance, Nasr City Cairo Egypt

**Keywords:** COVID‐19, learning, medical, online, perception, students

## Abstract

**Background and Aims:**

The viral agent of the novel coronavirus disease 2019 (COVID‐19) continues to spread worldwide, leading to a global pandemic. this may negatively affect students' mental health who have to maintain their learning efforts. Therefore, we aimed to assess students' perceptions of the online learning programs designed for university students in Arab countries during the COVID‐19 pandemic.

**Methods:**

This cross‐sectional study was conducted on university students using a self‐administered online questionnaire in 15 Arab countries, including 6779 participants. The actual sample size was calculated using the EpiInfo program calculator. The validated, piloted questionnaire assessed the effectiveness of internet‐based distance learning applications used in these countries during the pandemic. The SPSS version 22 was used.

**Results:**

Among the 6779 participants, 26.2% believed that their teachers diversify learning methods, 22.0% thought that their teachers were able to treat the weakness the students have, and 30.7% agreed that their teachers efficiently communicate with them through COVID‐19 internet‐based learning process. Around 33% of students participated in lectures effectively, 47.4% submitted their homework within accepted deadlines, and 28.6% thought that their colleagues did not cheat during exams and homework. Around 31.3% of students believed that online‐based learning had a role in directing them towards research, and 29.9% and 28.9%, respectively, believed that online learning had a role in developing analytical thinking and synthesis skills. Participants reported many suggestions to enhance the process of internet‐based distance learning in the future.

**Conclusion:**

Our study suggests that online‐based distance learning in Arab countries still needs more improvement as students still are more inclined toward face‐to‐face teaching. However, exploring the factors that influence students' perceptions of e‐learning is vital for improving the quality of online‐based distance learning. We recommend exploring the perceptions of educators regarding their experience towards online‐based distance learning during COVID‐19 lockdown.

## 1 INTRODUCTION

The viral agent of the novel coronavirus disease 2019 (COVID‐19) continues to spread worldwide, leading to a global pandemic. Many countries have launched social distancing measures to limit the propagation of the disease. However, these measures profoundly impact all aspects of human life, including education.^1^, ^2^, ^3^, ^4^ According to United Nations Educational, Scientific and Cultural Organization (UNESCO), most educational establishments, from primary schools to universities, have suspended face‐to‐face learning.^5^ However, this may negatively affect students' mental health who have to maintain their learning efforts. A Chinese study revealed that the COVID‐19 death count negatively impacted general sleep quality among college students. It also showed that the pandemic affected negative emotions indirectly through sleep quality.^6^ A previous study documented a significant association between anxiety among college students and delays in academic activities.^7^ This proves that one of the most critical challenges in this era of social distancing is maintaining the learning process and ensuring its flexibility. There were many efforts to promote E‐learning resources, and some guidance tools have been released to ensure the high quality of the process.^8^, ^9^ For instance, the Ministry of Education in China has innovated “Disrupted classes, Undisrupted Learning,” which aims to provide flexible online learning programs to more than 270 million students.^9^ Another study conducted in Libya on 3348 medical students showed that there are acceptable levels of knowledge, attitudes, and practice of e‐learning. the majority of the respondents disagreed that e‐learning could be used easily there.^10^

Nevertheless, the transition to online programs has brought many changes within the quality of the teaching tools and methods and the possibilities of changing the learning routine. Moreover, these innovations may be a significant burden for Arab students since their educational system is mainly based on face‐to‐face interaction since open educational resources are still limited in many Arab schools and universities.^8^

Therefore, this cross‐sectional was conducted to assess the perspectives of university students regarding internet‐based distance learning that has been adopted in Arab countries during this pandemic era, which supports future workforce and give us a lesson how to deal with the new COVID‐19 challenge.

## 2 METHODS

### 2.1 Study design, study population, and sampling

This cross‐sectional study was conducted to assess university students' perceptions regarding internet‐based distance learning during the COVID‐19 pandemic using a self‐administered online questionnaire. The study was conducted over a 2‐month duration (April and May 2020).

The validation of the questionnaire was performed through three main phases, first of all, face validation was conducted by sending the questionnaire to multiple experts for their feedback, second, the scale used inside the questionnaire was assessed for internal consistency, and which result was 0.93, and finally, a pilot sample size of 5% of the total sample was collected from each participating country (20 samples from each country) in which participants were asked about the clarity of the questionnaire, their comments were taken into consideration.

The study included university students from the following 15 Arab countries: *Algeria, Bahrain, Egypt, Iraq, Jordan, Lebanon, Libya, Morocco, Oman, Palestine, Qatar, Saudi Arabia, Syria, Tunisia, and Yemen*. Both Sudan and Kuwait were excluded since the policy of both countries was to pause the education process rather than starting online learning. Besides, Djibouti, Comoros, and Somalia were excluded due to Arabic language barriers, and the United Arab Emirates and Mauritania were excluded due to lack of communication.

A pre‐validated pre‐piloted questionnaire was distributed to a convenience sample of university students in each country. The actual sample size was calculated using the EpiInfo program calculator. Each cohort of students consisted of approximately 400 individuals from each country. In this survey, the recruited students were selected from various disciplines, including Health Sciences, Natural Sciences, Engineering, and Humanities Sciences. We used STROBE guidelines to report the study.

### 2.2 Questionnaire development and data collection

The questionnaire consisted of three main parts. The first part included demographic data (age, year of study, gender, marital status, country, university type, academic field, and level). The second part assessed the internet‐based distance learning applications used in these countries during the pandemic. The third part assessed the students' perceptions of teachers' performance, students' performance, and the role of internet‐based distance learning on the intellectual level learned. The scale's reliability was tested using Cronbach's alpha test using the statistical package for social science (SPSS) software version 22, resulting in 0.93, which is considered reliable. Content validity was performed by contacting educational specialists and researchers. The last part was about the suggested recommendations to enhance the internet‐based distance learning process in the future. Before data collection, a pilot sample was collected from all participating countries; the total pilot sample was 291, and content validity was asked from each applier, the feedback was taken into consideration. The survey link was available on the Google platform, and the university students were asked to complete the questionnaire. It did not require the participant to log in before filling the survey to ensure anonymity and protect data confidentiality. The process did not gather IP addresses, web cache, or cookies. The Google platform was used to store the data during the availability period, and the final data set was exported as a Microsoft Excel file.

### 2.3 Statistical analysis

Data were analyzed using SPSS software version 22 (SPSS Inc. The descriptive analysis was done using mean and SD for continuous variables. The categorical variables were presented as frequencies and percentages.

### 2.4 Ethical approval and consent to participate

The study has been conducted in alignment with the known Ethical research and surveillance recommendations for emergencies and disasters. The study protocol was approved by the ethical committee at the Institutional Review Board of Jordan University of Science and Technology (Approval number 2020‐PHA‐9). Consent to participate was obtained from the participants via the survey as the research used an online platform. All participants were fully informed about the study's nature and benefits on the first page of the survey.

## 3 RESULTS

**Figure 1:**
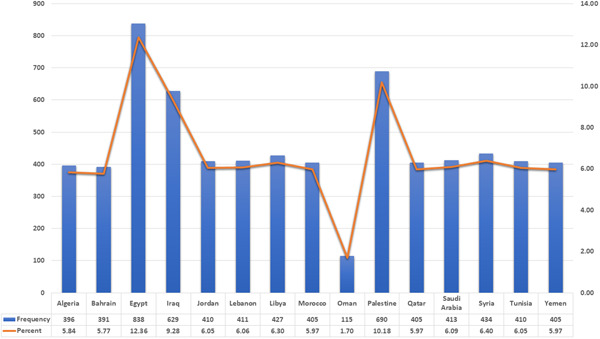
Students’ distribution according to their countries (*n* = 6779).

The questionnaire was filled out by 6779 bachelor's degree university students from 15 different Arab countries. The average age was 21.9 (SD = 3.4). Of the included participants, 4084 (60.2%) were females, 5314 (78.4%) were living in rural settings, 4734 (69.8%) were enrolled in a public university, and 1891 (27.9%) were enrolled in a private university. Most of the included participants (91.9%) were single. The field of study varied among participants; 3486 (51.4%) were involved in health science faculties, 1028 (15.2%) were natural science students, and 937 (13.8%) were engineering students. The socio‐demographic characteristics of the study sample are presented in Table 1. The distribution of the included participants among countries is shown in Figure 1.

**Table 1:** Socio‐demographic characteristics of the study sample (*n* = 6779).

Age	Mean (SD)	21.9 (3.4)
Year of study, *n* (%)	Second‐year or below	2781 (41.1)
	Third–fourth year	2632 (37.9)
	Fifth‐year or higher	1366 (15.7)
Gender, *n* (%)	Male	2695 (39.8)
	Female	4084 (60.2)
Marital status, *n* (%)	Single	6229 (91.9)
	Married	503 (7.4)
	Divorced	31 (0.5)
	Widow	16 (0.2)
Residency, *n* (%)	Rural	5314 (78.4)
	Urban	1465 (21.6)
University type, *n* (%)	Private	1891 (27.9)
	Public	4734 (69.8)
	International	154 (2.3)
Field of study, *n* (%)	Health science	3486 (51.4)
	Natural Science	1028 (15.2)
	Engineering	937 (13.8)
	Humanities	1328 (19.6)

**Figure 2:**
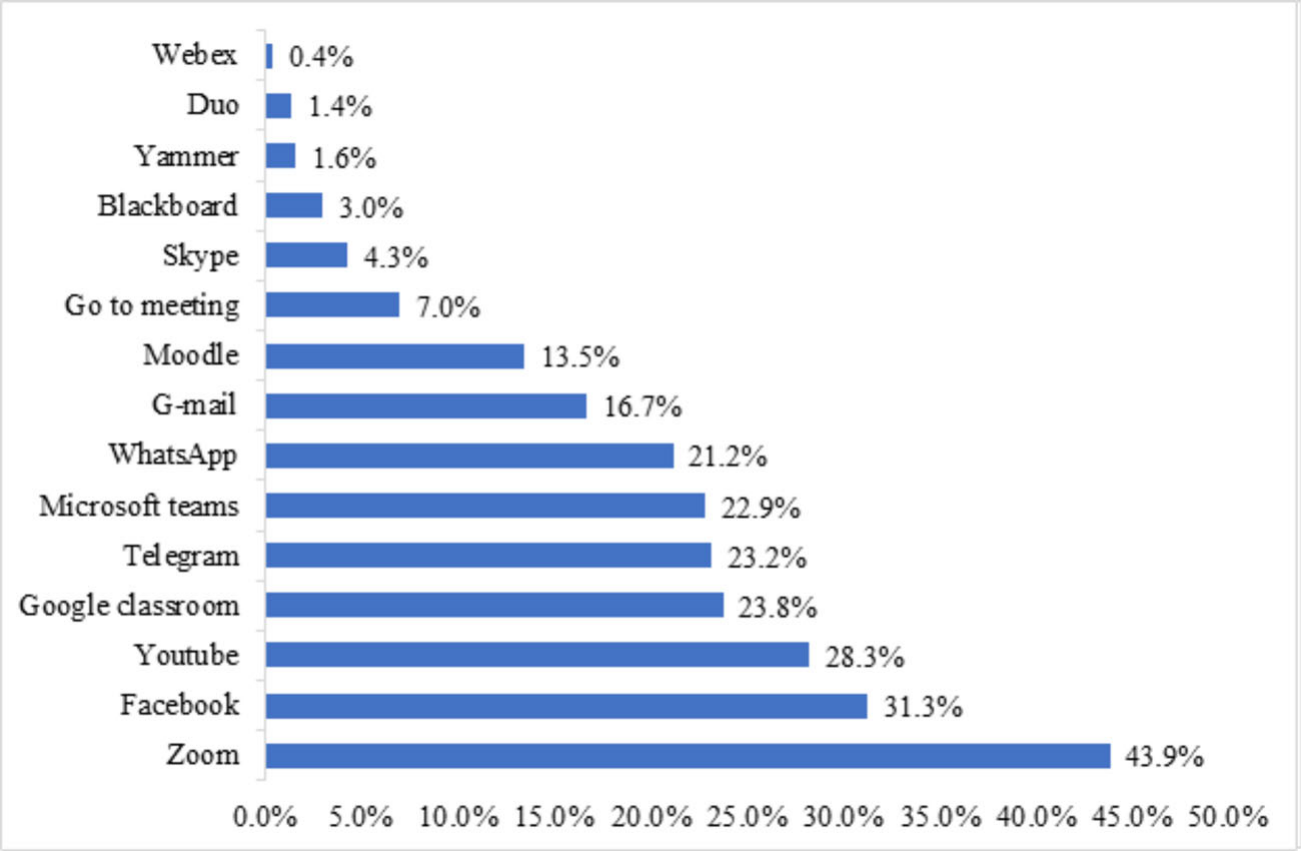
Most commonly used application during COVID‐19 Internet‐based distance learning (*n* = 6779). COVID‐19, coronavirus disease 2019.

When assessing the most common application using during the pandemic, Zoom was the most commonly used application (*n* = 2976, 43.9%), followed by Facebook (*n* = 2122, 31.3%), YouTube (*n* = 1918, 28.3%), and Google classroom (*n* = 1613, 23.8%), Figure 2.

Only a small percentage of students believed that their teachers showed good performance during COVID‐19 internet‐based distance learning. Only 26.2% (*n* = 1779) of the included students believed that their teachers diversify learning methods, and only 22.0% (*n* = 1493) thought that their teachers were able to treat the weakness the students have through the internet‐based distance learning period. Around 30.7% of the students (*n* = 2078) agreed that their teachers efficiently communicate with them through COVID‐19 internet‐based distance learning process. All items regarding students' perceptions of teachers' performance during COVID‐19 internet‐based distance learning are presented in Table 2.

**Table 2:** Students’ perceptions towards teacher performance during COVID‐19 internet‐based distance learning, (*n* = 6779).

Statement	Strongly disagree	Disagree	Neutral	Agree	Strongly agree
Teachers diversify learning methods, *n* (%)	1606 (23.7)	1511 (22.3)	1883 (27.8)	1117 (16.5)	662 (9.8)
Teachers diversify testing and homework's methods, *n* (%)	1692 (25.0)	1421 (21.0)	1650 (24.3)	1293 (19.1)	723 (10.7)
Teachers determine criteria for homework's evaluation, *n* (%)	1785 (26.3)	1381 (20.2)	1817 (26.8)	1167 (17.2)	629 (9.3)
Teachers treat the weaknesses students have, *n* (%)	2523 (37.2)	1518 (22.4)	1245 (18.4)	931 (13.7)	562 (8.3)
Teachers efficiently communicate with students, *n* (%)	1582 (23.3)	1340 (19.8)	1779 (26.2)	1332 (19.6)	746 (11.0)
Teachers consider students’ psychological status, *n* (%)	2799 (41.3)	1182 (17.4)	1298 (19.1)	850 (12.5)	650 (9.6)
Teachers consider academic differences between students, *n* (%)	2401 (35.4)	1423 (21.0)	1435 (21.2)	967 (14.3)	553 (8.2)

Abbreviation: COVID‐19, coronavirus disease 2019.

Students also provided their self‐perception about overall students' performance during the COVID‐19 internet‐based distance learning (Table 3). Around one‐third of them (*n* = 2262) agreed/strongly agreed that students participated in lectures effectively, and 47.4% of the students (*n* = 3211) reported that they were able to submit their homework and projects within accepted deadlines. Only 28.6% (*n* = 1940) of the participating students thought that their colleagues did not cheat during exams and home works.

**Table 3:** Students’ perceptions towards students’ performance during COVID‐19 pandemic internet‐based distance learning, (*n* = 6779).

Statement	Strongly disagree	Disagree	Neutral	Agree	Strongly agree
Students participated effectively in lectures, *n* (%)	1416 (20.9)	1224 (18.1)	1877 (27.7)	1441 (21.3)	821 (12.1)
Students submitted homework and projects within accepted deadlines, *n* (%)	944 (13.9)	914 (13.5)	1710 (25.2)	1875 (27.7)	1336 (19.7)
Students used extra references besides basic references, *n* (%)	1077 (15.9)	926 (13.7)	1723 (25.4)	1728 (25.5)	1325 (19.5)
Students did not cheat during exams and home works, *n* (%)	2151 (31.7)	1115 (16.4)	1573 (23.2)	1095 (16.2)	845 (12.5)

Abbreviation: COVID‐19, coronavirus disease 2019.

Regarding the role of internet‐based distance learning on the intellectual level learned, 31.3% of participants (*n* = 2120) thought this learning process had a role in directing students toward scientific research. Also, 29.9% (*n* = 2024) believed that internet‐based distance learning had a role in developing analytical thinking between students, and 28.9% (*n* = 1962) assumed that it might contribute to developing synthesis and discussion skills between students, as shown in Table 4.

**Table 4:** Students’ perceptions towards the role of internet‐based distance learning during COVID‐19 pandemic on their intellectual level (*n* = 6779).

Statement	Strongly disagree	Disagree	Neutral	Agree	Strongly agree
Internet‐based distance learning had a role in directing students toward scientific research, *n* (%)	1723 (25.4)	1252 (18.5)	1684 (24.8)	1354 (20.0)	766 (11.3)
Internet‐based distance learning had a role in developing analytical thinking between students, *n* (%)	1750 (25.8)	1317 (19.4)	1688 (24.9)	1325 (19.5)	699 (10.3)
Internet‐based distance learning had a role in directing students toward innovation, *n* (%)	1965 (29.0)	1213 (17.9)	1594 (23.5)	1316 (19.4)	691 (10.2)
Internet‐based distance learning had a role in developing synthesis and discussion skills between students, *n* (%)	1895 (28.0)	1322 (19.5)	1600 (23.6)	1289 (19.0)	673 (9.9)
Internet‐based distance learning considers technical ability differences between students, *n* (%)	2019 (29.8)	1179 (17.4)	1456 (21.5)	1131 (16.7)	994 (14.7)

Abbreviation: COVID‐19, coronavirus disease 2019.

**Figure 3:**
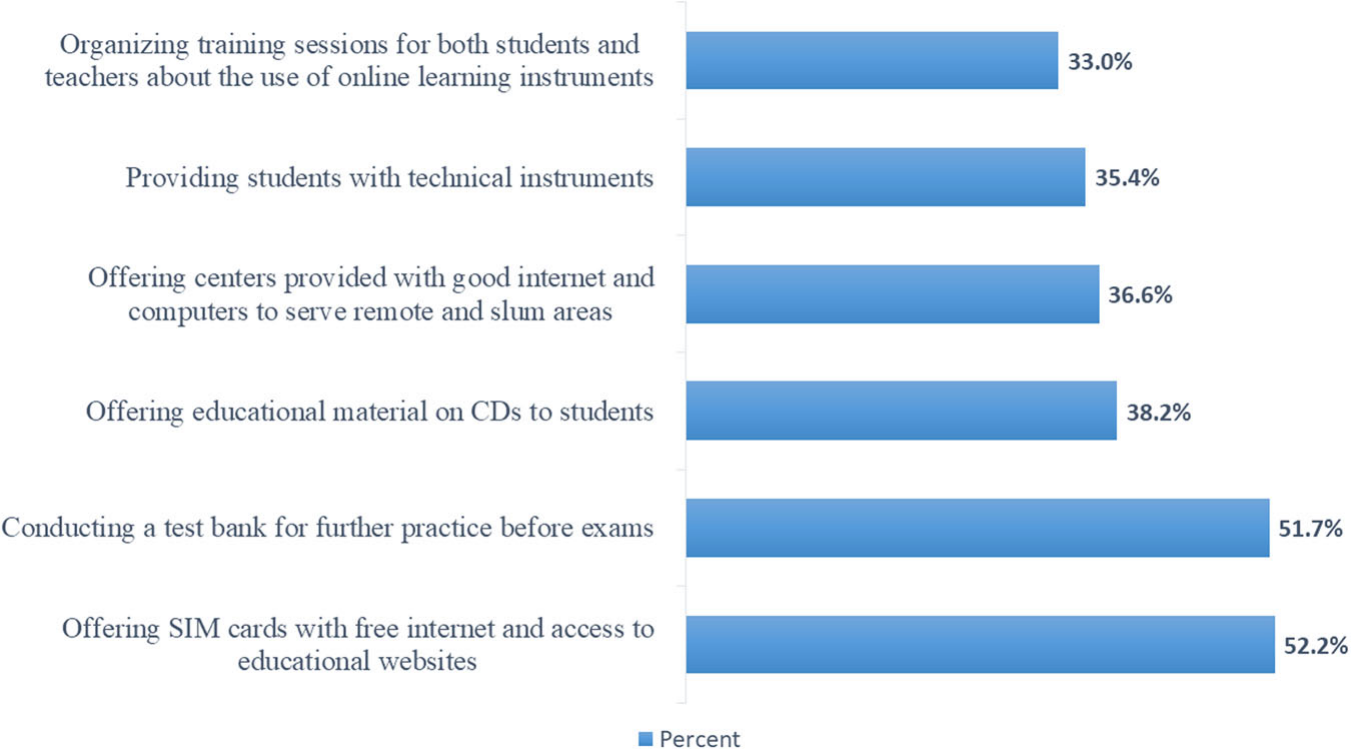
Participants’ suggestions to enhance internet‐based distance learning in the future (*n* = 6779).

Participants reported many suggestions to enhance the process of internet‐based distance learning in the future (Figure 3).

The main solutions suggested by students were to provide SIM cards with free internet and access to educational websites (*n* = 3538, 52.2%) and conducting a test bank for further practice before exams (*n* = 3503, 51.7%). Other solutions were given, including offering educational material on CDs to students (*n* = 2590, 38.2%), providing internet centers provided in remote and slum areas (*n* = 2482, 36.6%), providing students with technical instruments (*n* = 2401, 35.4%), and organizing training sessions for both students and teachers about the use of online learning instruments (*n* = 2238, 33.0%).

## 4 DISCUSSION

In this study, attempts have been made to discuss the effectiveness of online‐based distance learning programs that have been implemented for undergraduate university education in 15 Arab countries amid the COVID‐19 era. The accelerated spread of the COVID‐19 pandemic led to a worldwide lockdown that forced educational institutions to close their campuses and shift to online learning. Many institutions did not have comprehensive infrastructures to utilize a fully functional online classroom, so they were obligated to rely on online third‐party services to carry out their education modules; hence, online platforms such as Zoom, Facebook, and others were reported high in our study. Still, none of the available open‐access platforms provide a perfect option for online learning. The “perfect” mixture might not even be possible. As described in their article, Daniel and Marquis challenged distance educators to provide the right mixture between independent study and interactive learning activities and strategies.^11^, ^12^ Bates. et al. described characteristics for a successful educational application or technology and introduced the ACTIONS model (Access, Costs, Teaching and learning, Interactivity, Organizational issues, Novelty, and Speed).^13^

In our study, the Zoom platform was the most commonly used learning tool during COVID‐19 internet‐based distance learning; hence, the Zoom platform can provide us hints on how a perfect online‐based distance learning tool could be. Zoom is a teleconference service that provides a real‐time audio‐visual learning experience, with supportive tools for moderators to view participants, control engagement, record meetings with auto‐rendering, options of large audience capacity, options for private classroom creation. It is available on all common personal devices (computers and smartphones). Zoom also provides an option to create meetings with limited time and limited capacity, which are free of charge, making it affordable. Unfortunately, news spread regarding Zoom security concerns.^14^ Implementation of Zoom was carried out at Michigan State University (MSU). In their experience study, users reported reduced costs as well as it is simple, ease of use, and stable connection with the internet.^15^

In our study, through the internet‐based distance learning process, 30.7% of students agreed that their teachers efficiently communicate with them, 26.2% agreed that their teachers diversify learning methods, and 22.0% agreed that their teachers could treat the weakness the students have.

Based on a study reported by Anderson,^16^ if any of the three levels of interaction (student–student, student–teacher, and student–content) is assumed at an outstanding level, development of sufficient levels of meaningful and deep learning is warranted. In our study, students believe their commitment to deadlines was markedly high by submitting homework on time. Moreover, student exposure to extra learning resources was enhanced. The results of this study were consistent with a previous report by Demuyakor,^17^ which showed that students are satisfied with the learning resources available, with a mean of 3.74 out of 5.

However, nothing comes without doubts and downfalls; our study represents low overall teachers' performance using internet‐based distance learning. This is consistent with a previous study conducted in a private medical college that reported that more than 70% of students showed negative perceptions about e‐learning.^18^ Students believe that online‐based distance learning significantly encouraged cheating, which probably is related to lack of the usually controlled exam settings, the inexperience of educators, and exam answers being only one click away. The effect of online‐based distance learning on the intellectual level of students was not agreed upon, as students did not seem to be encouraged to engage in scientific research, develop analytical thinking, innovation, nor developing a synthesis of discussion skills. Unfortunately, online‐based distance learning does not discriminate between students' technical abilities.

Suggested solutions for effective engagement with online‐based distance learning included training staff and conducting a pretest for students to be familiar with online learning assessment tools. Additionally, engagement with online‐based distance learning platforms can be enhanced by providing technical support (computers and internet connection) for all parties involved and providing free access to websites and platforms used to commence online‐based distance learning. Cheating prerequisite a development of cheat‐proof exams and assignments that require more profound levels of processing, which eventually can lead to deepening students' memories.^19^

A meta‐analysis by the US Department of Education in 2010 using 50 contrasts of high‐quality internet‐based versus face‐to‐face courses showed a consistent overall advantage in student learning from internet‐based higher education.^20^ Success or failure of online‐based distance learning experience cannot be solely judged by the findings of our study, as several other than students' perspectives should be further investigated, and the development of objective assessment tools for online‐based distance learning will provide a more comprehensive view of its value.

### 4.1 Limitations

Because of COVID‐ 19 situation, the data collection process was done using online survey distributed social media in students' groups, which could have been unable to reach students who don't have access to social media platforms. We published the survey through various online channels to improve its visibility among the students and to reduce sampling bias. The questionnaire contained self reported questions that lead to recall bias.

## 5 CONCLUSION

This study highlighted students' perceive to educational transition during COVID‐19. Our findings suggest that online‐based distance learning in Arab countries still needs more improvement as students still are more inclined toward face‐to‐face teaching. However, exploring the factors that influence students' perceptions of e‐learning is vital for improving the quality of online‐based distance learning. We recommend exploring the perceptions of educators regarding their experience towards online‐based distance learning during the COVID‐19 lockdown.

## AUTHOR CONTRIBUTIONS

**Nael Kamel Eltewacy**: Formal analysis; methodology; project administration; software; supervision; validation. **Rana K. Abu Farha**: Project administration; supervision. **Sajeda G. Matar**: Formal analysis; investigation; methodology; software; validation; writing—original draft. **Afnan W. M. Jobran**: Data curation; formal analysis; visualization; writing—original draft. **Nesma Gebril Nagi**: Conceptualization; resources; writing—original draft. **Amira Yasmine Benmelouka**: Conceptualization; data curation; formal analysis; methodology; writing—original draft. **Hagar Ismail Rohim**: Resources; writing—original draft. **Enas M. Yasseen**: Methodology; writing—original draft. **Omnia Ayman**: Resources; visualization. **Aya N. El‐Din Sayed**: Methodology; writing—original draft. **Reem Ahmed M. Abdallah**: Data curation; formal analysis. **Rana Ahmed M. Abdallah**: Conceptualization; writing—review & editing. **Alaa M. Yousef**: Data curation; formal analysis. **Ahmad B. Yahia**: Investigation; methodology. **Muhamad Zakaria Brimo Alsaman**: Conceptualization; methodology; software; writing—review & editing. **Mahmoud A. Ebada**: Formal analysis; methodology; project administration; writing—review & editing.

## EARG COLLABORATORS

Jaffer Taha Al‐timimi^1^; Sarah Mahdi Shalash^2^; Mahmut Armağan^3^; Mohamed Abdel‐Razzaq^4^; Mawadda Abdullah Al‐Kumati^5^; Eman A. El‐Masry^6,7^; Abdullah Ahmed Alharbi^8^; Mohamed Aweis Abdulkadir^9^; Safaa M. Hmady^10^; Mariam Kanaan^11^; Mays Ahoubani^12^; Esra'a yahya bakier^13^; Nesrine Ben Hadj Dahman^14^; Abdullah Ahmed Areqi^15^; Ali hassan salah al‐hadi'^16^; Oumaima Outani^17^; EL MOUHI Hinde^18^; Sara Aldali^19^; Salam Ibrahim Abou Safrah^20^; Raghad Khaled Mohammad Saeed^21^; Nadia bounoua^22^; Ranim Naoum^23^; Mustafa Mohamed Ismaeel^24^; Asmaa M. Elaidy^25^; Mohammed Saad Akrawi^26^; Ramy Ashraf Attia^27^; Malak Ayad Al‐tahan^28^

^1^Iraq University of Kufa‐ College of Medicine

^2^University of Baghdad/College of Science/Department of Biology Iraq

^3^Kahramanmarash Sutcu Imam University, Faculty of Medicine, Department of Internal Medicine

^4^Al‐Darbak, Faculty of Medicine University, Tripoli, Libya

^5^Faculty of Human Medicine, University of Tripoli, Libya

^6^Department of Pathology, Microbiology and Immunology Unit, College of Medicine, Jouf University, Saudi Arabia

^7^Department of Medical Microbiology and Immunology, College of Medicine, Menoufiya University, Egypt

^8^Taif University, Faculty of Medicine, Saudi Arabia

^9^Ain Shams University, Faculty of Medicine

^10^Beirut Arab University, Faculty of Medicine Lebanon

^11^American University of Beirut Faculty of Health Sciences Lebanon

^12^PharmD, Faculty of Pharmacy, University of Jordan, Jordan

^13^Faculty of Pharmacy, Jordan University of Science and Technology Jordan

^14^University of Tunis El Manar, Faculty of Medicine of Tunis. Head of Research Department, Doctors of the World, Tunisia

^15^Department of Pharmacology, Faculty of Pharmacy, University of Science and Technology, Hodiadah, Yemen

^16^The Hoddidah University” Faculty of Clinical Pharmacy' Yemen

^17^Faculty of Medicine and Pharmacy of Rabat, Mohammed V University

^18^Center for Doctoral Studies Engineering Sciences and Techniques, Faculty of Sciences and Technologies, Medicinal and Translational Research, Faculty of Medicine and Pharmacy, University Sidi Mohammed Ben Abdellah of Fez, Morocco

^19^College of Pharmacy, Qatar University, Doha, Qatar

^20^College of Pharmacy, QU Health, Qatar University, Doha, Qatar

^21^Qatar University, College of Health Sciences, Department of Public Health, Health Education Qatar, Doha

^22^Department of Exact Sciences, Faculty of Science and Technology, Normal Higher School of Bechar, Algeria

^23^Faculty of Medicine, University of Oran 1, Oran 31000, Algeria

^24^Cairo University, Kasr Al‐ainy, Faculty of Medicine, Egypt

^25^MD Psychiatry, Lecturer at Psychiatry Department, Faculty of Medicine for Girls, Al‐Azhar University, Egypt

^26^Faculty of Pharmacy, National University of Science and Technology, Muscat, Oman

^27^Faculty of Pharmacy, National University of Science and Technology, Muscat, Oman

^28^Faculty of Pharmacy, National University of Science and Technology, Muscat, Oman

## CONFLICT OF INTEREST STATEMENT

The authors declare no conflicts of interest.

## ETHICAL STATEMENT

The study has been conducted in alignment with the known Ethical research and surveillance recommendations for emergencies and disasters. The study protocol was approved by the ethical committee at the Institutional Review Board of Jordan University of Science and Technology (Approval number 2020‐PHA‐9). Consent to participate was obtained from the participants via the survey as the research used an online platform. All participants were fully informed about the study's nature and benefits on the first page of the survey.

## TRANSPARENCY STATEMENT

The lead author Nael Kamel Eltewacy affirms that this manuscript is an honest, accurate, and transparent account of the study being reported; that no important aspects of the study have been omitted; and that any discrepancies from the study as planned (and, if relevant, registered) have been explained.

## Data Availability

All data generated or analyzed during this study are included in this article (Tables, Figures, and Supplementary data). Original data set/raw data are available from the corresponding author on reasonable request.
